# Patients experience with preoperative use of anti-obesity medications and associations with bariatric surgery expectations

**DOI:** 10.1016/j.soard.2024.08.041

**Published:** 2024-09-14

**Authors:** Jason M. Samuels, Mayur B. Patel, Christianne L. Roumie, Wesley Self, Luke Funk, Matthew D. Spann, Kevin D. Niswender

**Affiliations:** a Section of Surgical Sciences, Division of General Surgery, Vanderbilt University Medical Center, Nashville, Tennessee; b Department of Surgery, Veterans Administration Tennessee, Nashville, Tennessee; c Department of Medicine, Valley VA Health Care System Geriatric Research Education Clinical Center (GRECC), Nashville, Tennessee; d Department of Medicine, Vanderbilt University Medical Center, Nashville, Tennessee; e Vanderbilt Institute for Clinical and Translational Research, Vanderbilt University Medical Center, Nashville, Tennessee; f Department of Emergency Medicine, Vanderbilt University Medical Center, Nashville, Tennessee; g Department of Surgery, University of Wisconsin-Madison, Madison, Wisconsin; h Department of Surgery, William S. Middleton Veterans Health Administration Hospital, Madison, Wisconsin

**Keywords:** GLP-1 receptor agonists, Bariatric surgery, Anti-obesity medications, Obesity, Weight loss

## Abstract

**Background:**

Few studies have investigated the use of anti-obesity medications (AOMs) before bariatric surgery and how prior use impacts patients’ goals and expectations for surgery.

**Objectives:**

This study investigated associations between patients’ experiences with AOMs and weight loss expectations before bariatric surgery.

**Settings:**

Single tertiary university hospital.

**Methods:**

Patients were electronically surveyed with a 31-item questionnaire via email or the patient portal with a primary predictor variable of AOMs presurgery. Outcomes included degree of weight loss and weight regain and motivation for seeking surgery.

**Results:**

A total of 346 persons were invited to complete the survey; 112 surveys (32.4%) were completed, with 7 excluded because of not answering the AOM question. 73% reported AOM use. Among those who took AOMs before seeking bariatric surgery, average weight loss was 13 kg (SD 10) corresponding to a 4.4-kg/m^2^ decrease in BMI. Of past AOM recipients, 87% reported weight regain on stopping AOMs. Average weight regain was 18 kg (SD 13; 126% increase). Patients reported improved longevity and quality of life as motivation for seeking surgery, with AOM use history having no effect. Subjects reported an average weight loss goal of 65.8 kg (39% of baseline weight) from bariatric surgery.

**Conclusions:**

AOMs were commonly used in those seeking bariatric surgery, but motivation for surgery did not differ by AOM use history. Motivations were most often related to goals for better overall health.

As the therapies targeting obesity expand, patients’ experiences with weight loss treatments continue to evolve. Several medications for weight loss were approved by the Food and Drug Administration (FDA) in the last 5 years, including the latest generation of glucagon-like peptide 1 receptor agonists (GLP1RA) that achieve 15%–20% total weight loss (TWL) [1,2]. Most recently, in 2023 the FDA approved tirzepatide, a glucose-dependent insulinotropic polypeptide (GIP)/GLP1RA di-agonist, based on the results of the SURMOUNT 1 trial in which participants lost on average 21% of their body weight. Building on the interest in prior GLP1RA therapies approved for weight loss such as semaglutide, the resulting FDA approval of tirzepatide for weight loss received considerable media coverage [3,4]. As a result, these latest generation of anti-obesity medications (AOMs) with GLP1RAs and GIP/GLP1RAs represent the fastest growth in prescribing of all pharmaceuticals and represent a landmark change in the approach to obesity treatment [5,6].

Despite these pharmaceutical advances, bariatric surgery remains the most effective treatment, with TWL averaging 25%–35% of presurgical body weight [7,8]. Prior studies demonstrate that patients’ expectations for weight loss after surgery exceed the average achieved with surgical weight loss alone [9]. Cohn reported that patients seeking surgical weight loss are motivated by a desire to improve overall health or quality of life, rather than to achieve a particular weight [10]. These studies largely pre-date the use of medications specifically developed and approved for treating obesity. Thus, little is known about the frequency of use of AOMs before bariatric surgery, or how the growth of AOM use has impacted patients’ motivations and expectations for bariatric surgery.

Our aim was to describe the frequency of AOMs before seeking surgical weight loss. We also sought to evaluate the association between patients’ experience with AOMs and weight loss expectations before bariatric surgery, the motivations for seeking surgical weight loss, and desired weight loss.

## Methods

### Study design and survey instrument

This single-center, cross-sectional survey study was conducted at a university medical center surgical weight loss clinic between December 13, 2023, and March 13, 2024. The survey instrument consisted of 31 items (Appendix 1).

Domains included demographics, AOMs used, motivation for seeking weight loss treatment, and items regarding combination weight loss strategies. Patients were asked to rank their selected motivatons on a scale of 1 to 6 with 1 being least important and 6 being most important. Remaining items were multiple choice, Likert scale, and free text responses. Completion of the survey required 5–10 minutes, and participants did not receive compensation for completion. The institutional review board deemed this study exempt from IRB review (IRB 232013), and consent was implied with completion of the questionnaire.

### Study population

We identified eligible participants from the clinic schedule if they were listed as a “new consult” patient who was seeking bariatric surgery, age ≥18 years, English speaking, and had an active patient portal account or email on file. Patients seeking both primary and revisional bariatric surgery were included. Exclusion criteria included those patients listed as “do not contact” for research studies and those who did not answer the item regarding prior AOM use.

### Instrument administration and data collection

The instrument was administered electronically via the patient portal application, a patient-centered mobile app with messaging capabilities, or via email. The message contained information about the survey and a link to complete the survey electronically. All participants received a reminder message to complete the survey within 1 week of the initial message. No participant data was collected beyond the information participants provided within the survey instrument.

### Predictor variable: AOM

The primary predictor was patient-reported usage of AOMs at any point before their visit to the surgical weight loss clinic. Any use, as determined by the patient, was considered as an exposure to AOMs. A complete list of medications, including generic and brand names, can be found in Appendix Table 1. Among those who used AOMs, we describe their self-reported weight loss and weight regain.

### Outcomes: motivation for weight loss and desired weight loss goal

We compared the association between patients’ motivations for undergoing bariatric surgery and goals among those with and without use of prior AOM. Possible motivations used in the survey instrument were derived using commonly reported motivations from previously published literature [11–14]. The secondary outcomes included patients’ self-reported desired weight loss from surgery and differences in responses depending on prior GLP1RA exposure.

### Statistical analysis

Data were collected using REDCap [15]. Descriptive statistics were used to report survey responses. Categorical variables were presented as frequency and percentage, and continuous variables were presented as median and interquartile range or means and standard deviation as appropriate. Medians (with interquartile range) and percentages were compared using the Wilcoxon-Mann-Whitney test, χ^2^ test, or Fisher exact test as appropriate. A 2-sided *P* value of <.05 was considered statistically significant. Statistical analyses were conducted using GraphPad Prism Version 10.1.1 (Boston, MA).

## Results

### Study cohort and demographics

A total of 346 patients were invited to participate (279 by MHAV patient portal message and 67 by email), and 112 responded (32%). Response rates differed between those contacted by patient portal versus email (36% versus 16%). Seven patients were excluded for nonresponse on the AOM item, providing a final cohort of 105 respondents. Median age of respondents was 48 years (IQR 42–59 years) with 77% of the cohort identifying as female. Respondents were predominantly White (63%) followed by Black (20%), “other” (2%), and American Indian, Native Hawaiian, or other Pacific Islander (1.1% each). Eighteen patients (17%) reported having previously undergone bariatric surgery (Table 1). Seventy-three percent reported having previously taken AOMs, and of those 74% reported trialing more than 1 AOM agent in the past.

Patients reported an average weight loss of 14.2 kg (±10.9 kg) with prior AOM use (median BMI loss with medications was 4.4 kg/m^2^). Sixty-five (87%) subjects reported weight regain after stopping the medication (2 nonrespondents), with average weight regain of 18.0 kg (±12.9 kg, 126% of original weight loss).

### Participants’ weight loss preference

In response to the item “If weight loss were the same, which approach to losing weight would you prefer to take?” 43% selected surgery, 23% selected medications, and 33% were uncertain or had no preference. The majority of patients (73%) reported a “moderate” or “complete” willingness to take medications to enhance weight loss after surgery. Acceptance decreased to 58% if lifelong medication was required, or if the medication was a weekly injection (59% “moderate” or “completely” willing).

Participants were also asked, “In which of the following scenarios would you be willing to take a weight loss medicine after bariatric surgery if the medicine is given as an injection taken weekly at your home?” Most respondents noted a relatively small amount of additional weight loss was necessary to consider such therapies. Participants expressed the greatest interest in adjuvant AOMs if they offered 20 lb (35%) or 30 lb (36%) additional weight loss. There were 30% who noted 40 lb, 17% for 50 lb and 19% for ≥60 lb of additional weight loss as needed to take an injectable AOM after surgery.

### Weight loss motivations and expectations

**Fig. 1.**
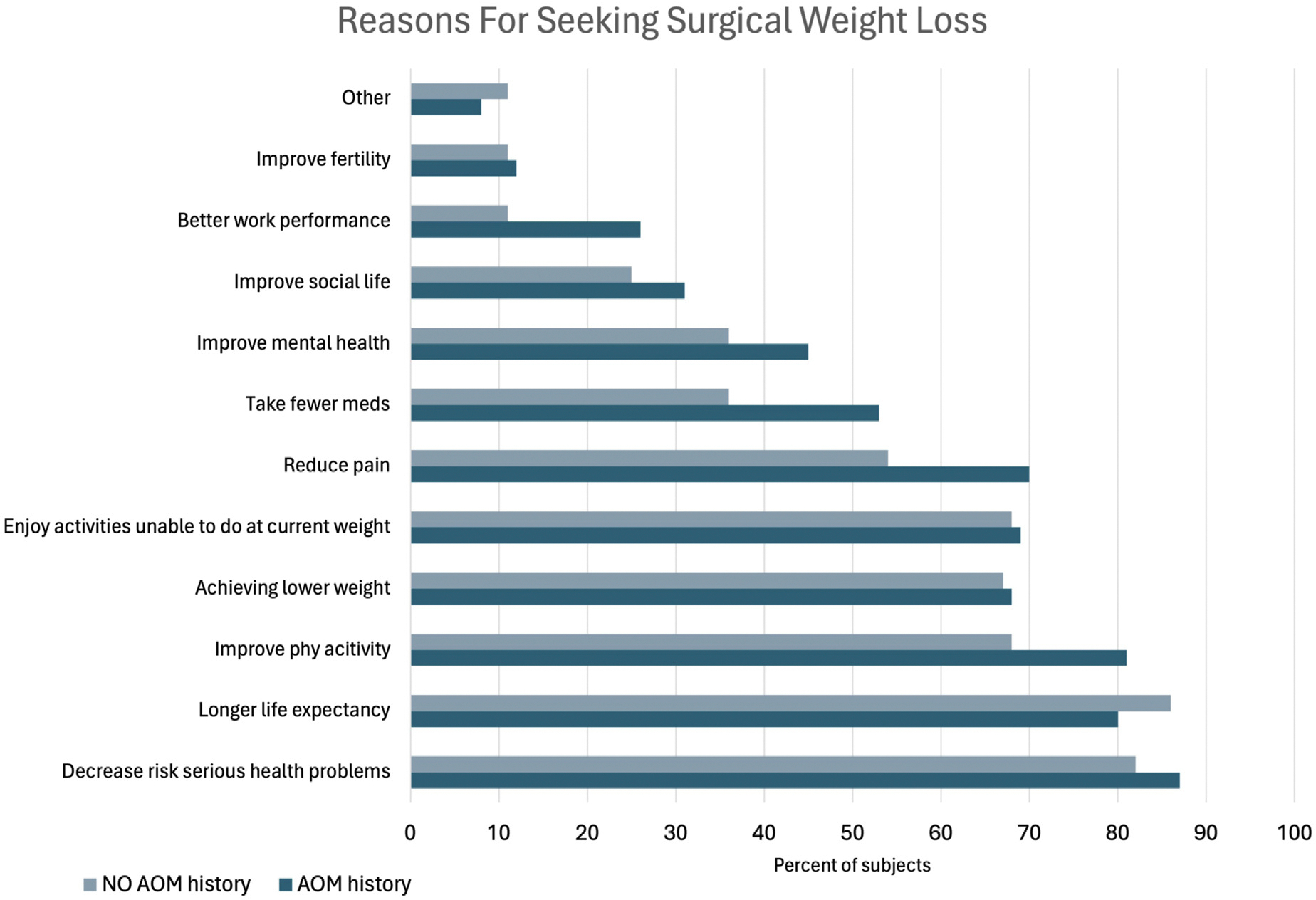
Comparison between patients with and without history of anti-obesity medication usage of selected motivations for seeking bariatric surgery.

Participants were asked to select their motivations for weight loss from a list of common reasons. Decreasing the risk of serious health problems was the most common answer (91%) followed by longer life expectancy (87%) and improved physical activity (82%). Achieving a lower weight was similarly, but not the most, common (73%). When comparing participants with or without prior history of AOM usage, there was no difference between groups in the frequency of noted motivations for seeking surgical weight loss (all *P* > .1, Fig. 1). We also compared how the 2 groups rated the importance of individual motivations. Only a desire to reduce pain differed in importance between groups, with patients who had no prior history of AOM usage rating pain reduction with greater importance than those with no AOM usage history (median 4 versus 2, *P* = .003). We similarly investigated how prior GLP-1 use impacted motivations for surgery, finding no difference in the motivations for seeking surgical weight loss. Both respondents with and without a history of GLP-1 use ranked the most important motivations for seeking surgery as decreasing risk of serious health conditions, followed by achieving longer life expectancy and improving physical activity capabilities.

We asked participants, “How much weight (in pounds) do you (or did you) expect to lose with surgery?” The median response was 65.8 kg (IQR 56.7–77.7). This was on average 39% (IQR 34%–47%) expected total body weight loss from bariatric surgery. Expected weight loss from bariatric surgery was similar among participants with or without a history of AOM use (68 versus 45 kg; 40% TWL versus 37% TWL, *P* = .2). We compared patients with GLP-1 use history to those without in terms of expected weight loss from surgery, with similar estimates of weight loss in both groups (42.5% versus 39.7%, *P* = .4).

## Discussion

We report a significant majority of patients have previously used AOMs before seeking surgical weight loss, with most patients trialing more than 1 AOM. The primary reason for seeking weight loss was most often to increase longevity and quality of life and was not associated with prior AOM use. Participants more often reported a preference for surgical weight loss compared with medical treatments but were willing to use AOMs to enhance weight loss after bariatric surgery.

The impact of preoperative weight loss, including through mandated medical weight loss programs, have been extensively investigated with minimal demonstrated benefit [16]. Interestingly, incorporation of AOMs as part of these preoperative weight loss programs are rare. This likely owes to the fact that although preoperative weight loss is encouraged, and occasionally even required before surgery, payers rarely cover AOMs as preparation for surgery [16]. The minimal existing literature on preoperative AOM use primarily details case reports of attempts at preoperative weight loss in patients in which at their current weight, bariatric surgery carries prohibitive risk [17,18]. Our findings suggest that despite this absence of evidence guiding AOM use before surgical weight loss, use of AOMs prior to bariatric surgery appears to occur in a majority of patients seeking surgical weight loss. The majority of survey respondents reported use of AOMs before surgery and on average trialed 2 medications.

Several prior studies examined motivations and goals for weight loss as well, and our findings match closely with the current literature with some notable differences [10,14]. In congruence with our study, prior publications have found improved overall health as the most common reason for seeking bariatric surgery [11,19,20]. However, in contrast to our findings, Ahlich et al. found only 10% of patients reported improved longevity as a motivating factor for seeking surgical weight loss [19]. Hult et al.’s findings more closely matched our own, with weight loss, improved comorbidities, and longevity as the 3 most common motivators for weight loss, respectively, in a multinational, all-female study [12]. To our knowledge, this is the first study to compare motivations for seeking bariatric surgery between patients who have and have not previously used AOMs. With the exception of patients’ desires to improve their physical activity, the 2 groups did not differ in stating the importance of the most common reasons listed for seeking surgical weight loss.

Our survey further reinforced the concept of expectations for surgery-induced weight loss exceeding the average weight loss achieved with sleeve gastrectomy and gastric bypass. This aligns with the current literature with expected excess weight loss of 72%–106% [21–24]. In our results, we report that patients were willing to use injectable AOM after surgery to achieve additional desired weight loss. Ultimately, more studies are needed to investigate the clinical and psychosocial benefits of enhanced weight loss with the use of adjuvant medical therapies.

This study has several important limitations to consider. First, the sample of participants are those who have failed to achieve weight loss with AOMs and are interested and seeking bariatric surgery. Given that we did not have access to a nonsurgical comparator group, the sample likely over-represents those who failed medical treatments and those who have a positive view toward surgery. Second, this study was conducted at a single surgical weight loss clinic with the respondent population predominantly White and female, which may limit the generalizability. Third, the response rate for the survey was 32% of those contacted. Nonresponse bias may have influenced the results we report. We do not believe the sample of respondents were differential in their reported AOM usage before bariatric surgery, weight loss experiences, and how these experiences may have affected motivations for seeking bariatric surgery. We did not have respondents expand on the duration of AOM use or ask about respondents compliance with AOMs. However, the average degree of weight loss reported suggests respondents used AOMs consistently and for a significant length of time. Lastly, we recognize that larger studies enrolling a more diverse sample from both medical and surgical clinics would add to and confirm these findings.

Several questions remain that future studies can address. Future surveys should investigate how access to and tolerance of AOMs may influence patients’ interest in or desire to undergo bariatric surgery. Additionally, future studies should investigate how patients seeking different bariatric surgical options (e.g., sleeve versus gastric bypass) may differ in their motivations to undergo surgery. Lastly, more research is needed into how patients’ motivations for surgery differ depending on the presence of varying preexisting comorbidities.

## Conclusion

AOMs are used by a majority of patients before seeking surgical weight loss, with most patients using more than 1 AOM before considering surgery. With minimal data guiding AOMs in patients who are candidates for bariatric surgery currently, future studies should explore how use of these medications can be optimized to improve weight loss outcomes and enhance the effectiveness of surgery. Patients also continue to desire greater weight loss than is offered by surgery on average, and patients in our study expressed significant interest in using AOMs after surgery to further enhance weight loss. Prospective studies and clinical trials are needed to determine the most effective way AOMs can be implemented after bariatric surgery.

## Supplementary Material

Appendix 1

Appendix Table 1

Supplementary material associated with this article can be found, in the online version, at http://doi.org/10.1016/j.soard.2024.08.041.

## Figures and Tables

**Table 1:** Baseline demographics for overall cohort and the cohorts with history of AOM usage and without AOM usage

Variable (respondents per question)	Overall (n = 105)	No AOM use (n = 28)	Prior AOM use (n = 77)

Age (n = 85), median (IQR)	48 (42–59)	56 (44–60)	46 (41–57)
Missing, n (%)	20 (19)	6 (21)	14 (18)
Sex/gender (self-identified sex) (n = 97), n (%)			
Male	13 (12)	6 (21)	7 (9)
Female	81 (77)	19 (68)	62 (81)
Nonbinary	1 (1)	0	1 (1)
Other	1 (1)	0	1 (1)
Missing	9 (9)	3 (11)	6 (8)
Race (n = 92), n (%)			
White	66 (63)	19 (68)	47 (61)
Black	21 (20)	2 (8)	19 (25)
American Indian	1 (1)	1 (4)	0
Native Hawaiian or other Pacific Islander	1 (1)	1 (4)	0
Preferred not to answer	1 (1)	1 (4)	0
Other	2 (2)	0	2 (3)
Missing	14 (13)	4 (14)	10 (13)
Ethnicity (n = 89), n (%)			
Hispanic	3 (3)	2 (7)	1 (1)
Non-Hispanic	84 (80)	22 (79)	62 (81)
Prefer not to answer	1 (1)	0	1 (1)
Missing	17 (16)	4 (14)	13 (17)
Anthropometrics			
Weight, kg (n = 97), median (IQR)	118 (106–141)	116 (108–135)	120 (101–145)
Missing, n (%)	9 (9)	3 (11)	6 (8)
Height, cm (n = 92), median (IQR)	165 (160–173)	168 (163–177)	164 (160–170)
Missing, n (%)	13 (12)	4 (14)	9 (12)
BMI, kg/m^2^ (n = 92)*, median (IQR)	44 (39–52)	40 (37–45)	45 (40–53)
Missing, n (%)	13 (12)	4 (14)	9 (12)
Prior bariatric surgery (n = 105), n (%)	18 (17)	4 (14)	14 (18)
Missing, n (%)	0	0	0
Type of surgery (n = 18), n (%)			
Sleeve gastrectomy	7 (39)	1 (25)	6 (43)
Gastric bypass	2 (11)	1 (25)	1 (7)
Gastric band	5 (28)	2 (50)	3 (21)
Revisional surgery	4 (22)	0	4 (29)
Missing	0	0	0

Medication used (n = 77)			n (%)

Bupropion-naltrexone (Contrave)			9 (12)
Liraglutide			3 (4)
Metformin			39 (51)
Orlistat			11 (14)
Phentermine			45 (58)
Phentermine-topiramate			15 (20)
Semaglutide			25 (33)
Setmelanotide			0
Tirzepatide			14 (18)
Topiramate			20 (26)
Other			16 (21)
Missing			0

AOM = anti-obesity medication; IQR = interquartile range; BMI = body mass index.

## References

[1] Jastreboff AM, Aronne LJ, Ahmad NN, Tirzepatide once weekly for the treatment of obesity. N Engl J Med 2022;387:205–16. 35658024 10.1056/NEJMoa2206038

[2] Wilding JPH, Batterham RL, Calanna S, Once-weekly semaglutide in adults with overweight or obesity. N Engl J Med 2021;384:989–1002. 33567185 10.1056/NEJMoa2032183

[3] FDA Approves Lilly’s Zepbound^™^ (tirzepatide) for Chronic Weight Management, a Powerful New Option for the Treatment of Obesity or Overweight with Weight-Related Medical Problems, PR Newswire; 2023.

[4] Gilbert D, Ovalle D. FDA approves lilly diabetes drug mounjaro for weight loss. Waltham, MA: Wash Post 2023. Available from: https://www.washingtonpost.com/health/2023/11/08/mounjaro-zepboundfda-approval-obesity/. Accessed March 1, 2024.

[5] Saxon DR, Iwamoto SJ, Mettenbrink CJ, Antiobesity medication use in 2.2 million adults across eight large health care organizations: 2009–2015. Obesity 2019;27:1975–81. 31603630 10.1002/oby.22581 PMC6868321

[6] Tichy EM, Hoffman JM, Tadrous M, National trends in prescription drug expenditures and projections for 2023. Am J Health Syst Pharm 2023;80:899–913. 37094296 10.1093/ajhp/zxad086

[7] Courcoulas AP, King WC, Belle SH, Seven-year weight trajectories and health outcomes in the longitudinal assessment of bariatric surgery (LABS) study. JAMA Surg 2018;153:427–34. 29214306 10.1001/jamasurg.2017.5025 PMC6584318

[8] Risstad H, Svanevik M, Kristinsson JA, Standard vs Distal Roux-en-Y gastric bypass in patients with body mass index 50 to 60: a double-blind, randomized clinical trial. JAMA Surg 2016;151:1146–55. 27626242 10.1001/jamasurg.2016.2798

[9] Aelfers SCW, Schijns W, Ploeger N, Patients’ preoperative estimate of target weight and actual outcome after bariatric surgery. Obes Surg 2017;27:1729–34. 28124235 10.1007/s11695-017-2556-2

[10] Cohn I, Raman J, Sui Z. Patient motivations and expectations prior to bariatric surgery: a qualitative systematic review. Obes Rev 2019;20:1608–18. 31419379 10.1111/obr.12919

[11] Jaensson M, Josefsson E, Stenberg E, Dahlberg K. Do reasons for undergoing bariatric surgery influence weight loss and health-related quality of life?-A Swedish mixed method study. PLoS One 2022;17:e0275868. 36215261 10.1371/journal.pone.0275868 PMC9550063

[12] Hult M, Te Riele W, Fischer L, Women’s reasons to seek bariatric surgery and their expectations on the surgery outcome - a multicenter study from five European countries. Obes Surg 2022;32:3722–31. 36151346 10.1007/s11695-022-06280-w PMC9613564

[13] Munoz DJ, Lal M, Chen EY, Why patients seek bariatric surgery: a qualitative and quantitative analysis of patient motivation. Obes Surg 2007;17:1487–91. 18219776 10.1007/s11695-008-9427-9

[14] Libeton M, Dixon JB, Laurie C, O’Brien PE. Patient motivation for bariatric surgery: characteristics and impact on outcomes. Obes Surg 2004;14:392–8. 15072662 10.1381/096089204322917936

[15] Harris PA, Taylor R, Minor BL, The REDCap consortium: building an international community of software platform partners. J Biomed Inform 2019;95:103208. 31078660 10.1016/j.jbi.2019.103208 PMC7254481

[16] Tewksbury C, Williams NN, Dumon KR, Sarwer DB. Preoperative medical weight management in bariatric surgery: a review and reconsideration. Obes Surg 2017;27:208–14. 27761723 10.1007/s11695-016-2422-7 PMC6060405

[17] Sari C, Seip RL, Umashanker D. Case report: off label utilization of topiramate and metformin in patients with bmI >/=50 kg/m(2) prior to bariatric surgery. Front Endocrinol 2021;12:588016. 10.3389/fendo.2021.588016 PMC7947603 33716960

[18] Martines G, Dezi A, Giove C, Efficacy of intragastric balloon versus liraglutide as bridge to surgery in super-obese patients. Obes Facts 2023;16:457–64. 37579738 10.1159/000531459 PMC10601677

[19] Ahlich E, Verzijl CL, Cunning A, Wright E, Rancourt D. Patient motivations and goals for bariatric surgery: a mixed methods study. Surg Obes Relat Dis 2021;17:1591–602. 34134941 10.1016/j.soard.2021.05.017

[20] Pearl RL, Wadden TA, Walton K, Health and appearance: factors motivating the decision to seek bariatric surgery. Surg Obes Relat Dis 2019;15:636–42. 30803880 10.1016/j.soard.2019.01.015 PMC6538485

[21] Fischer L, Nickel F, Sander J, Patient expectations of bariatric surgery are gender specific–a prospective, multicenter cohort study. Surg Obes Relat Dis 2014;10:516–23. 24951069 10.1016/j.soard.2014.02.040

[22] Price HI, Gregory DM, Twells LK. Weight loss expectations of laparoscopic sleeve gastrectomy candidates compared to clinically expected weight loss outcomes 1-year post-surgery. Obes Surg 2013;23:1987–93. 23794118 10.1007/s11695-013-1007-y

[23] Karmali S, Kadikoy H, Brandt ML, Sherman V. What is my goal? Expected weight loss and comorbidity outcomes among bariatric surgery patients. Obes Surg 2011;21:595–603. 20066502 10.1007/s11695-009-0060-z

[24] Heinberg LJ, Keating K, Simonelli L. Discrepancy between ideal and realistic goal weights in three bariatric procedures: who is likely to be unrealistic? Obes Surg 2010;20:148–53. 19789932 10.1007/s11695-009-9982-8

