# Supplementary material for: Patients experience with preoperative use of anti-obesity medications and associations with bariatric surgery expectations
Source: Surg Obes Relat Dis. Author manuscript; Available in PMC 2025 Jul 24. (PMC12288039; doi:10.1016/j.soard.2024.08.041)
Supplement: Appendix 1 [file NIHMS2094774-supplement-Appendix_1.pdf]

GRABS Survey

This is a research study being conducted at Vanderbilt University Medical Center by Dr. Jason Samuels, the study's principle investigator. The purpose of this survey is to learn from your experiences and thoughts about different ways to lose weight, such as surgery or medications. Your input will help us understand what people expect from weight loss surgery, whether doctors have suggested surgery, and if you've ever used medicines to lose weight before. We're also curious about your preferences between using medications or having surgery to lose weight. We'll ask about the reasons why losing weight is important to you and if you'd be interested in a study that offers a free weight loss injection after surgery. This information will help us care for our patients better by aligning medical treatments with patients goals and by helping us design studies to determine whether such additional therapy would be helpful at achieving more weight loss. Participation is entirely voluntary, answers are anonymous, and your decision to participate will in no way effect your care. By completing this survey, you provide your consent to pariticpate in this research study. If you have questions about this study you may contact Dr. Samuels via email at Jason.M.Samuels@VUMC.org or call his office at (615) 322 4504 ext 24504.

Have you previously undergone bariatric surgery?

◯ Yes
◯ No

If so, what bariatric surgery did you undergo?

☐ Sleeve Gastrectomy
☐ Gastric Bypass
☐ Gastric Band
☐ Unsure
☐ Other

Please specify which bariatric surgery you listed as other?

____

How much weight did you lose (in pounds) with bariatric surgery?

____

Did you experience any weight regain after bariatric surgery?

◯ Yes
◯ No

If yes, how much weight regain did you experience (in pounds)?

____

How much weight (in pounds) do you (or did you) expect to lose with surgery?

____

Has a healthcare provider (such as a primary care doctor) encouraged you to undergo bariatric surgery?

◯ Yes
◯ No
◯ Unsure

Questions about medications for weight loss.

Have you ever taken medicines for weight loss?

- ◯ Yes
- ◯ No

Which of the following medications have you tried for weight loss (select all that apply)?

- ☐ Bupropion-naltrexone (Contrave)
- ☐ Metformin
- ☐ Liraglutide (Saxenda)
- ☐ Orlistat (Xenical, Alli)
- ☐ Phentermine-topiramate (Qsymia)
- ☐ Semaglutide (Ozempic, Wegovy)
- ☐ Setmelanotide (Imcivree)
- ☐ Topiramate (Topamax)
- ☐ Tirzepatide (Mounjaro)
- ☐ Phentermine
- ☐ Other

What was the greatest number of pounds of weight that you lost with medicine?

____

Did you regain weight while taking or after stopping the medication?

- ◯ Yes
- ◯ No

How many pounds did you regain after taking the medicine?

____

Weight loss usage after bariatric surgery to enhance weight loss

If weight loss were the same, which approach to losing weight would you prefer to take:

- ◯ Taking medicine daily or weekly
- ◯ Undergoing surgery
- ◯ No preference
- ◯ Unsure

Please state how willing you would be to take a weight loss medicine after bariatric surgery in the following scenarios, from not at all willing to completely willing:

	Not at all willing	A little willing	Somewhat willing	Moderately willing	Completely willing
How willing would you be if you could increase how much weight you lost with surgery by taking a medication after surgery?	○	○	○	○	○
How willing would you be if you had to take the medication after surgery for the rest of your life?	○	○	○	○	○
How willing would you be if the medication was a weekly injection into your skin?	○	○	○	○	○

Reasons for seeking weight loss treatments

People have different reasons for wanting to lose weight. The list below presents some reasons. Please select all the reasons listed below that motivate you to lose weight (select up to six choices).

- ☐ Achieving a lower weight
- ☐ Needing to take fewer medications
- ☐ Longer life expectancy
- ☐ Pain reduction
- ☐ Enjoying activities that I am not able to do at my current weight
- ☐ Decreasing my risk of serious health problems related to excess body weight
- ☐ Improved social life
- ☐ Improved physical activity
- ☐ Improved mental health
- ☐ Better work performance
- ☐ Improved fertility
- ☐ Other

Please describe your reason "other" for seeking weight loss treatment?

____

Of the previous selected reasons for desiring weight loss, please rank them in importance from least important to most important.

	1 Least Important	2	3	4	5	6 Most important
Achieving a Lower Weight	○	○	○	○	○	○
Needing to take fewer medications	○	○	○	○	○	○
Longer life expectancy	○	○	○	○	○	○
Pain reduction	○	○	○	○	○	○
Enjoying activities that I am not able to do at my current weight	○	○	○	○	○	○
Decreasing my risk of serious health problems related to excess body weight	○	○	○	○	○	○
Improved Social Life	○	○	○	○	○	○
Improved Physical Activity	○	○	○	○	○	○
Improved Mental Health	○	○	○	○	○	○
Better Work Performance	○	○	○	○	○	○
Improved fertility	○	○	○	○	○	○
[other_wt_loss]	○	○	○	○	○	○

Questions regarding combined weight loss options.

How interested would you be in taking part in a study in which the weight loss injection (taken once a week) after surgery was provided at no cost for a year?	◯ Not at all interested<br>◯ A little interested<br>◯ Somewhat interested<br>◯ Moderately interested<br>◯ Completely interested
Would you like to be contacted about participating in such a study	◯ Yes<br>◯ No
Please provide your email address for further information regarding participating in these studies.	______________________________
In which of the following scenarios would you be willing to take a weight loss medicine after bariatric surgery if the medicine is given as an injection taken weekly at your home.	☐ If the medication led to 20 lbs more weight loss than surgery alone.<br>☐ If the medication led to 30 lbs more weight loss than surgery alone.<br>☐ If the medication led to 40 lbs more weight loss than surgery alone.<br>☐ If the medication led to 50 lbs more weight loss than surgery alone.<br>☐ If the medication led to 60+ lbs more weight loss than surgery alone.

General information

What is your age?

____

What is your sex

- ◯ Male
- ◯ Female
- ◯ Non-binary
- ◯ Other
- ◯ Prefer not to say

What is your race

- ☐ White/Caucasian
- ☐ Black/ African American
- ☐ Asian
- ☐ American Indian
- ☐ Alaska Native
- ☐ Native Hawaiian or other Pacific Islander
- ☐ Prefer not to say
- ☐ Other ____

Please specify your race you indicated as "other".

____

What is your ethnicity

- ◯ Hispanic
- ◯ Non-hispanic
- ◯ Prefer not to say

What is your current weight (in pounds).

____

What would you like your weight to be (in pounds).

____

What is your height in inches?

____
