# Supplementary material for: Patients experience with preoperative use of anti-obesity medications and associations with bariatric surgery expectations
Source: Surg Obes Relat Dis. Author manuscript; Available in PMC 2025 Jul 24. (PMC12288039; doi:10.1016/j.soard.2024.08.041)
Supplement: Appendix Table 1 [file NIHMS2094774-supplement-Appendix_Table_1.pdf]

Appendix Table 1
Medication
Buproprion-Naltrexone (Contrave)
Liraglutide (Saxenda)
Metformin
Orlistat (Xenical, Alli)
Phentermine
Phentermine-Topiramate (Qsymia)
Semaglutide (Ozempic, Wegovy)
Setmelanotide (Imcivree)
Tirzepatide (Mounjaro)
Topiramate (Topamax)
Other
